# President’s Address

**Published:** 1912-11-15

**Authors:** Chas. A. Tavel

**Affiliations:** Memphis, Tenn.


### PRESIDENT'S ADDRESS.

BY DR. CHAS. A. TAVEL, MEMPHIS, TENN.

Read before the Tennessee State Dental Society, June 6, 1912.

During the forty four years' existence of this Association, there have been quite as many addresses presented for your edification and consideration. Some have covered the history of our profession, ante-dating the time when the village blacksmith was called upon to relieve suffering humanity; some have recounted its advancement step by step; some have delt in generalities; some have touched upon scientific topics; some have peered into the future and foretold what has since been accomplished; while others looked backward to recall the men and the deeds of the past.

So, with such enlightenment by men, many of whom have been potent factors in the development of our profession in this state, and whose names and fame no doubt will go down in history as shining lights in a world-wide profession, it seems that there is left little or nothing about which to speak.

It is the supposition that a much greater civilization than ours perished in the darkness of the middle ages, and that thousands of years ago in Assyria, many of the wonderful contrivances which we call modern, were common. However, journeying through the valley of ages, the scientific and mechanical branches of dentistry have kept apace with that of other professions, though our science, with its leaps and bounds, has outstripped many of its associates.

With the knowledge obtained in recent years of infection with micro-organisms, we are no longer in doubt as to the cause of this or that ailment which we are called upon to combat. And by way of parenthesis, permit me to say that the names of Lister and Pasteur should be emblazoned upon the memory of each and every one of us. It was Pasteur who advanced the theory that all septic infections are due to micro-organisms, and it was Lister who applied the surgical pathology and demonstrated on the human subject that destructive inflammation and suppuration following a surgical operation, are caused by germs present, either in the air, in the tissue of the patient, or the hands of the operator, or on his instruments. We then learned the cause of caries and lesions of the oral cavity. This brought about the concentration of our thoughts and study on Oral Hygiene and Prophylaxis, which subject has by pen and voice, and I might say by clinical skill received more attention in the past few years than all others combined.

The mighty wave of oral Hygiene which began a comparatively short time ago, has continued to spread to such an extent that it is not now confined to any particular community, state, or nation, but has covered the length and breadth of the universe.

While the philanthropists and generous-hearted laymen have established institutions and laboratories, the scientists are busily engaged in working to the end that a much better health condition be brought about. And it may truthfully be said that dentistry is receiving its share of consideration.

The Educational Boards in many states have adopted physiological text-books, in which are inserted chapters pertaining to the teeth and surrounding tissues. Our own state is not in the rear while this battle for progress is being fought. Already we have secured the co-operation of our State Text Book Commission, and a chapter, especially prepared by members of this Association, entitled "Care of the Teeth," has been added to our school Physiology. Our Legislative Committee is wide-awake, and we may expect to see in the near future a law requiring the services of dental examiners for all schools throughout the state.

Now while we have the public's interest aroused, we should not lose sight of the fact that the work before us is yet tremendous, and if we fall short of our undertaking and let this wave recede, the finger of derision will surely be pointed toward us.

While much consideration has been given to the subject of public education regarding the advantages to be derived from the teaching of Oral Hygiene, and while we are imbued with the idea of the actual necessity of this form of dental teaching when we come to the question of what particular method to use in bringing this work to the attention of the greatest number of people, many and varied suggestions might be offered. There has developed in the art of photography, the motion picture, which has become the greatest educational medium known to modern science. It is by means of the picture that we are enabled to first to teach the child to discern one object from another. It is the picture, the object itself which conveys to the mind the most vivid impression. Words are only names, but the pictures represent the things themselves. They entertain and instruct one while making a vivid and lasting impression Motion pictures are used to show how food is grown and prepared, how various articles are manufactured, how the house fly multiplies and spreads infectious matter, and is now being used to great advantage in the teaching of arts and sciences. An effort was made in this state as well as other places, to work up a picture along dental lines which could be used to advantage in lectures and work before student bodies, mothers' clubs, etc., but in every case the expense was found to be so great that their efforts were temporarily abandoned. However, with renewed activity on the part of the National Mouth Hygiene Association, which in turn submitted the idea and outlined to the National Dental Association the plan was not only commended, but their aid was at once offered to futher the development of the proposition. We regret that we could not secure a duplicate film of this picture to present at this meeting, but it will be a few more weeks before it is completed. The expense of the original film will be about $2,000, which will be paid by the National Hygiene Association out of its funds derived from membership fees. It is proposed to make duplicate films from this and have each state purchase one at the actual cost of production, which will be between $250 and $300. The prevailing idea is that this film shall go from place to place in each state, being shown one day and one night in each picture theatre. In this way the greatest number of people will be reached in the least possible time. I would recommend that this association be one among the first to secure a film, and that an assessment be made, or some other plan adopted by which the necessary amount can be raised for this purpose.

The plan of re-organization recommended for several years by preceding presidents, and which has been this year put into effect, and also more liberal appropriations not only from our association, but from other sources, are essential if we expect to continue this good work. We should have a representation so strong throughout the state, that when any measure is brought before the Legislature pertaining to the welfare of our profession, there should not be a doubt as to its passage.

While we have men of integrity, energy and enthusiasm, who have sacrificed their time and money toward educating the public in oral hygiene and prophylaxis, we must admit that the number engaged in the work is too small, and the compensation is not sufficient. There should be taken from the school, or some other fund, the necessary amount to carry on the work of our Oral Hygiene Committee, and this Association should also set aside annually a sufficient amount necessary to keep our Committee on Re-organization actively engaged. Ohio has gone forward in this Oral Hygiene movement, as is shown by an appropriation of over $500.00 to its Oral Hygiene Committee, in 1910; last year it gave the same amount, and added $100.00 to the work of the National Committee, and this year it has gone still further in its liberal appropriations. New Jersey has set a precedent that would be wise for us to follow. It has placed upon its statute books a law which, if it could be made uniform would be of incalculable benefit to every state in the Union. It is as follows:—

Whenever any Dental Association regularly incorporated under the laws of this State shall maintain and conduct in any city of this State a dental clinic, or clinics, where indigent persons, residents of such city may receive treatment and relief without charge or fee therefor, it shall be lawful for the board or body having control of the finances of such city, to appropriate and pay such Association each year such sum, or sums not exceeding in all the sum of Five Thousand Dollars, as it shall beem advisable, to be used and applied by such Association only for the support, maintenance and equipment in such city of a dental clinic, or clinics, for the free treatment of indigent persons, residents of such city, and for no other purpose whatever.

When we see the signal victories that are being won by our northern, eastern, western, and extreme southern co-laborers, we cannot restrain a feeling of anxiety to push forward the great work that stands before us with outstretched hand beckoning us on. We need closer affiliation and co-operation among the profession, which would result in a general wiping out of the petty jealousies that seem to exist in our ranks.

The opinion seems to prevail that the State Boards of Dental Examiners should be more closely associated and co-operate with one another in securing laws for the betterment of our profession. We should have a uniform reciprocity law in vogue in every nook and corner over which flies the American Flag. There are a few states in which this law is recognized, but others seem slow to grasp the importance of it. Kentucky has recently had passed a law which gives its Dental Board the power to issue a certificate to practice dentistry to any dentist applying for registration without examination, other than clinical, its only requirements being that he or she must be of good moral character, a legal, competent and ethical practitioner of dentistry, and come from a state whose requirements are equal to that of hers, provided said certificate is presented within six months from date of issue. Likewise its Board issues certificates to those who leave for another state. Illinois has reciprocity with Indiana, Iowa, Kansas, Michigan, Nebraska, Ohio, Wisconsin and Minnesota, so there is no reason why it should not be made to include every state in the Union.

I would suggest that a committee on co-operation be created to foster closer relationship between the members of the Medical and Dental Professions. Essays on the different branches of dentistry should be of as much benefit to them as theirs are to us.

We should also see to it that a member of this association is placed on the State Board of Health, and that the State Board of Education and City Superintendents and teachers of schools should co-operate with us and have dental lectures at all their meetings. I trust you will devise some effective means by which these recommendations can be put into effect.

It is with a feeling of much gratification that I report to you that as the result of an enactment of Congress previous to our meeting last year, of a law entitled "A Bill to Improve the Status and Efficiency of the Dental Surgeons in the U. S. Army," an examination was recently held and twenty nine appointments were made as acting dental surgeons of the army with the ranks of First Lieutenant after three years' service. Other vacancies are being filled, and a full quota of sixty-six may soon be looked for. This is a bit of timely legislation, as it shows the stamp of Congress' approval of our efforts toward obtaining a higher standard.

At our last meeting there was created a standing committee to be known as the Benevolent Committee, consisting of three members. Their terms of Office are to be one, two and three years respectively. One vacancy occurs each year, which place is to be filled by election of another member. The duties of this committee are to work up an interest in the state for benevolent purposes, to collect and take charge of all funds for this cause, and to disburse same on order of the Association. This enactment is especially intended to provide for the aged and infirm members of our profession. No specified plans were outlined in regard to devising ways and means to raise the fund. The National Association at its meeting last year adopted a similar plan, and is now endeavoring to secure a uniform action of the various Associations. It is proposed to have every member of the various societies participate in this great benevolent work, which would be the means of bringing cheer and happiness to those of our ranks who are in distress and suffering for the necessities of life. It is estimated that between twenty and thirty thousand dollars can be procured annually by an assessment of one dollar per year, to say nothing of the voluntary contributions and donations. I would therefore recommend that this Association have its constitution and by-laws so changed as to provide an assessment, or change its dues from two to three dollars per year for this purpose.

The committee on re-organization, appointed by the president, has been making arrangements to organize component societies throughout the state, but as yet no great headway has been made, but as reforms move slowly, we should not feel discouraged, but lend a helping hand by soliciting those whom we know to be eligible to join our Association. I would recommend that this committee be continued for an indefinite period in order to give its members an opportunity to pursue the course they have outlined. I wish to thank the various committees for the work they have done, and trust this meeting will go down in our history as one of the most instructive and enjoyable ever held.

As we enter upon another year's work no one can say what it will bring forth. We start out with a blank page, but ere we meet again, upon it many lines will be written. Some will convey the sad intelligence of final departures from our ranks, while others will impart the news of some achievement which will perhaps startle the nation. Then let us so live by being just to ourselves, and just to others, that we may bring about a more efficient professional service, and a more generous reward for our efforts.

